# A 1-bp deletion in bovine *QRICH2* causes low sperm count and immotile sperm with multiple morphological abnormalities

**DOI:** 10.1186/s12711-022-00710-0

**Published:** 2022-03-07

**Authors:** Maya Hiltpold, Fredi Janett, Xena Marie Mapel, Naveen Kumar Kadri, Zih-Hua Fang, Hermann Schwarzenbacher, Franz R. Seefried, Mirjam Spengeler, Ulrich Witschi, Hubert Pausch

**Affiliations:** 1 Animal Genomics, Institute of Agricultural Sciences, ETH Zürich, Universitätstrasse 2, 8092 Zürich, Switzerland; 2 Clinic of Reproductive Medicine, Vetsuisse Faculty, University of Zürich, Winterthurerstrasse 260, 8057 Zürich, Switzerland; 3 ZuchtData EDV Dienstleistungen, Dresdner Straße 89/B1/18, 1200 Wien, Austria; 4 Qualitas AG, Chamerstrasse 56, 6300 Zug, Switzerland; 5 Swissgenetics, Meielenfeldweg 12, 3052 Zollikofen, Switzerland; 6 Present Address: Genome Biology of Neurodegenerative Diseases, Deutsches Zentrum Für Neurodegenerative Erkrankungen e. V. (DZNE), Otfried-Müller-Str. 23, 72076 Tübingen, Germany

## Abstract

**Background:**

Semen quality and insemination success are monitored in artificial insemination bulls to ensure high male fertility rates. Only ejaculates that fulfill minimum quality requirements are processed and eventually used for artificial inseminations. We examined 70,990 ejaculates from 1343 Brown Swiss bulls to identify bulls from which all ejaculates were rejected due to low semen quality. This procedure identified a bull that produced 12 ejaculates with an aberrantly small number of sperm (0.2 ± 0.2 × 10^9^ sperm per mL) which were mostly immotile due to multiple morphological abnormalities.

**Results:**

The genome of this bull was sequenced at a 12× coverage to investigate a possible genetic cause. Comparing the sequence variant genotypes of this bull with those from 397 fertile bulls revealed a 1-bp deletion in the coding sequence of the *QRICH2* gene which encodes the glutamine rich 2 protein, as a compelling candidate causal variant. This 1-bp deletion causes a frameshift in translation and a premature termination codon (ENSBTAP00000018337.1:p.Cys1644AlafsTer52). The analysis of testis transcriptomes from 76 bulls showed that the transcript with the premature termination codon is subject to nonsense-mediated mRNA decay. The 1-bp deletion resides in a 675-kb haplotype that includes 181 single nucleotide polymorphisms (SNPs) from the Illumina BovineHD Bead chip. This haplotype segregates at a frequency of 5% in the Brown Swiss cattle population. Our analysis also identified another bull that carried the 1-bp deletion in the homozygous state. Semen analyses from the second bull confirmed low sperm concentration and immotile sperm with multiple morphological abnormalities that primarily affect the sperm flagellum and, to a lesser extent, the sperm head.

**Conclusions:**

A recessive loss-of-function allele of the bovine *QRICH2* gene likely causes low sperm concentration and immotile sperm with multiple morphological abnormalities. Routine sperm analyses unambiguously identify homozygous bulls for this allele. A direct gene test can be implemented to monitor the frequency of the undesired allele in cattle populations.

**Supplementary Information:**

The online version contains supplementary material available at 10.1186/s12711-022-00710-0.

## Background

Semen quality and male fertility are complex traits with low to moderate heritabilities [1]. These traits are routinely monitored in thousands of bulls as a service from the semen collection centers to the cattle breeding industry to ensure high success rates in artificial insemination. The availability of large cohorts with repeated semen quality analyses facilitates the separation between genetic and environmental factors and the differentiation between female and male factors that contribute to variation in reproduction [2]. Such phenotypes are ideally suited to characterize the genetic architecture of male fertility [3].

Genome-wide association testing revealed quantitative trait loci (QTL) for semen quality and male fertility in various breeds of cattle, many of them expressing non-additive effects [4–6]. The routine evaluation and recording of tens of thousands of ejaculates and millions of artificial inseminations also occasionally detect bulls with aberrant semen quality or strikingly low insemination success rates. Although diseases and environmental exposures can compromise male fertility [7], a drastically reduced semen quality in individual bulls often results from monogenic disorders [8–11]. Genome-wide case–control association testing helps to pinpoint genetic variants associated with such conditions. In this approach, genotypes at genome-wide markers are compared between affected bulls and an unaffected control group. Variants at which the genotypes differ between both groups are candidate causal variants that are subsequently subjected to further functional investigations.

Impaired semen quality and low fertility in healthy bulls can be caused by deleterious alleles at genes that show testis-biased or testis-specific expression [8–10]. Such alleles are spread disproportionately by females because they are typically not impacted by defective genes that are primarily expressed in the male reproductive system. Semen quality and male fertility are only evaluated in breeding bulls. Thus, recessive variants that compromise male reproduction can remain undetected in cattle populations for a long period of time or reach a high allele frequency without being detected [8].

Here we report a 1-bp deletion inducing a frameshift in the bovine *QRICH2* gene that, in the homozygous state, likely causes male infertility due to immotile sperm with multiple morphological abnormalities. Using historic and current semen quality data from two Brown Swiss bulls, we show that this mutation remained undetected although phenotypic consequences started to be observed almost two decades ago. Our findings offer the opportunity to implement direct gene testing to monitor the frequency of this undesired allele in cattle populations.

## Methods

### Animals and phenotypes

We assessed records for 70,990 ejaculates that were collected from 1343 Brown Swiss bulls between January 2000 and March 2018. All ejaculates were examined by laboratory technicians employed by Swissgenetics immediately after semen collection as part of routine quality assurance during semen processing. The parameters recorded were semen volume (mL), sperm concentration (million sperm per mL) that was quantified using photometric analysis, and the percentage of sperm with forward motility. The proportion of sperm with head and tail anomalies was quantified for each ejaculate with scores ranging from 0 to 3 (0: no or very few anomalies, 1: less than 10% sperm with anomalies, 2: between 10 and 30% sperm with anomalies, 3: more than 30% sperm with anomalies). Subsequently, we considered 35,785 ejaculates that were collected from 1258 bulls aged between 330 and 550 days to identify those for which all ejaculates were discarded due to insufficient semen quality. Ejaculates that contained less than 300 million sperm per mL, less than 70% motile sperm, more than 10% sperm with head and tail abnormalities, or with a volume smaller than 1 mL were deemed to be unsuitable for artificial inseminations.

### Whole-genome sequencing and sequence variant genotyping

We extracted DNA from hair roots of a Brown Swiss bull that produced ejaculates with a low sperm count and immotile sperm with multiple morphological abnormalities (since the sequence read archive accession number is SAMEA6272098, this bull is hereafter referred to as SAMEA6272098). For whole-genome sequencing, an Illumina TruSeq DNA PCR-free paired-end library with a 400-bp insert size was sequenced on an Illumina NovaSeq6000 instrument. We used the fastp software [12] to remove adapter sequences, poly-G tails and reads that had a phred-scaled quality score less than 15 for more than 15% of the bases. Following quality control, we aligned 153,618,844 read pairs to the ARS-UCD1.2 assembly of the bovine genome using the mem-algorithm of the BWA software [13] with option -M to mark shorter split hits as secondary alignments and default settings for all other parameters. We marked duplicates using the Picard tools software suite (https://github.com/broadinstitute/picard). Alignments were sorted by coordinates using the Sambamba tool [14]. Sequencing depth was calculated using the mosdepth software (version 0.2.2, [15]) by considering only the reads with a mapping quality higher than 10.

The SAMEA6272098 bull and 521 cattle from various breeds (14 Hereford, 1 Nelore, 33 Grauvieh, 50 Fleckvieh, 2 Nordic Red Cattle, 47 Holstein, 243 Brown Swiss, 128 Original Braunvieh, and 3 Wagyu) were genotyped for sequence variants (single nucleotide polymorphisms (SNPs) and insertion-deletion mutations (indels)) using a multi-sample variant discovery and genotyping approach implemented with the HaplotypeCaller, GenomicsDBImport and GenotypeGVCFs modules from the Genome Analysis Toolkit (GATK, [16]). Subsequently, we applied best practice guidelines of the GATK for variant filtration and imputed missing genotypes using Beagle [17]. The reference-guided variant genotyping workflow applied here is described in detail in Crysnanto et al. [18]. Functional consequences of 41,659,308 autosomal variants were predicted according to the Ensembl (version 104) and Refseq (version 106) annotations of the bovine genome using the Variant Effect Predictor software [19] from Ensembl along with the SpliceRegion.pm plugin (https://github.com/Ensembl/VEP_plugins/blob/release/101/SpliceRegion.pm).

### Identification of candidate causal variants

We considered the whole sequences of 397 fertile bulls as controls to identify sequence variant genotypes associated with the sperm disorder of the SAMEA6272098 bull (see Additional file 1: Table S1). These 397 bulls were either key ancestor animals or artificial insemination bulls or both and as such were assumed to be fertile, although sperm quality was not examined for all of them. Assuming recessive inheritance of a deleterious allele, we screened the sequence variant genotypes of the SAMEA6272098 genome for homozygous sites that were never found in the homozygous state in fertile bulls. These variants were subsequently filtered to retain those with a predicted deleterious impact on a protein.

### Identification of haplotype carriers in the population

Imputed high-density genotypes of 33,045 cattle were used to assign the 1-bp deletion to a haplotype. Briefly, these 33,045 cattle were genotyped using different Illumina SNP microarrays [4]. Following quality control, the genotypes were imputed to a density of 683,903 SNPs with Beagle [20] using a reference panel of 1166 cattle that had BovineHD genotypes. We retained genotypes from 25,243 Brown Swiss and 5228 Original Braunvieh animals from the Swiss herdbook populations. A subset of the genotyped animals (N = 285) also had whole-genome sequence-derived genotypes.

We inspected the phased (and partially imputed) BovineHD genotypes of 285 animals which also had the 1-bp deletion genotyped using whole-genome sequencing data. Specifically, we searched for a haplotype that extends on either side of the BTA19:55436705TC>T position of the deletion (BTA19 for *Bos taurus* chromosome 19) and that was shared in the heterozygous state by all sequenced animals carrying the 1-bp deletion. The genotypes and haplotypes of the 285 animals are in Additional file 1: Table S1. We used the alleles of the longest shared haplotype to identify heterozygous and homozygous haplotype carriers among 30,471 genotyped Brown Swiss and Original Braunvieh cattle. Alleles and positions of markers encompassed by the haplotype (see Additional file 2: Table S2) were also provided to Brown Swiss breeding associations and genetic evaluation centers to screen their genomic prediction reference populations and selection candidates for homozygous haplotype carriers.

### Whole transcriptome sequencing and read alignment

We used DNA and RNA sequencing data from a previously established expression QTL (eQTL) cohort to investigate transcript abundance and functional consequences caused by the 1-bp deletion. The eQTL cohort consisted of 76 mature bulls from which the testes were sampled at a commercial slaughterhouse after regular slaughter. Total RNA and DNA (see Additional file 3: Table S3) were extracted from testis tissue and prepared for sequencing as described earlier [21].

DNA samples were sequenced on an Illumina NovaSeq6000 sequencer using 150-bp paired-end libraries. Quality control (removal of adapter sequences and low-quality bases, and trimming of poly-G tails) of the raw sequencing data was carried out using the fastp software [12] with default parameters. Following quality control, between 70,493,763 and 307,416,205 read pairs per sample were aligned to the ARS-UCD1.2 version of the bovine reference genome [22] using the mem-algorithm of the BWA software (see above). Average coverage of the 76 DNA samples estimated using the mosdepth software (see above) ranged from 6.3 to 27.6 with a mean value of 12.6 ± 4.2. Sequence variant genotypes were called and filtered using the HaplotypeCaller, GenomicsDBImport, GenotypeGVCFs and VariantFiltration modules from the GATK as described above.

Total RNA sequencing libraries (2 × 150 bp) were prepared using the Illumina TruSeq Stranded Total RNA sequencing kit and sequenced on an Illumina NovaSeq6000 sequencer. Quality control (removal of adapter sequences and low-quality bases, and trimming of poly-A and poly-G tails) of the raw sequencing data was carried out using the fastp software [12]. Following quality control, between 191,160,837 and 386,773,085 filtered reads per sample (mean: 283,587,831 ± 43,284,185) were aligned to the ARS-UCD1.2 reference sequence and the Ensembl gene annotation (release 104) using STAR (version 2.7.9a) [23] with options -twopassMode Basic, -sjdbOverhang 100, -outFilterMismatchNmax 3, and -outSAMmapqUnique 60.

### Bioinformatic analysis

The structure of the bovine *QRICH2* gene (*ENSBTAG00000030173*, Gene-ID 530282) was analysed using data from Ensembl (version 104) and Refseq (version 106). Transcript abundance (in transcripts per million, TPM) was quantified for the 76 bulls of the eQTL cohort using kallisto [24] and aggregated to the gene level using the R package tximport [25]. Exon abundance was quantified using QTLtools [26]. Exon–exon junctions were visualized using the ggsashimi R package [27]. Read coverage as well as reference and alternative allele support at the 1-bp deletion were inspected using the Integrative Genomics Viewer (IGV, [28]). A binomial test was carried out with the R function *binom.test()* to examine if the alternate allele ratio in six heterozygous bulls was significantly different from 0.5.

### Genotyping of the 1-bp deletion

Polymerase chain reaction (PCR) and Sanger sequencing were used to validate the 1-bp deletion. PCR products from genomic DNA were amplified using the following primers: 5′-CATCGAGAAGGTGCAGATCC-3′ (forward) and 5′-CTGCCC ACCGTTTGTAGC-3′ (reverse) with a standard 50 µL PCR reaction mix (100 ng genomic DNA and final concentrations of 1× PCR reaction buffer, 200 µM dNTP mixture, 0.2 µM each primer and 0.05 units/µL JumpStart Taq Polymerase (Sigma)). The PCR amplification program consisted of an initial denaturation step at 94 °C for 30 s followed by 30 cycles of 94 °C for 10 s, 55 °C for 1 min, and 72 °C for 30 s with a final extension step at 72 °C for 1 min. All PCR products were purified with the GenElute PCR Clean-Up Kit (Sigma) and sent for Sanger sequencing (Microsynth, Switzerland). Sequencing results were analysed using the CLC Genomics Workbench software (Qiagen).

### Analysis of the semen from a bull homozygous for the 1-bp deletion

Ejaculates from a bull homozygous for the 1-bp deletion were collected at 14, 16, 17 and 20 months of age using an artificial vagina. Sperm concentration, total sperm count, and sperm motility were determined with an IVOS II CASA system (Hamilton Thorne Inc., Beverly, USA) using Leja 2-chamber slides (Leja, Nieuw-Vennep, the Netherlands). Semen was fixed in buffered formol saline solution (Na_2_HPO_4_ 4.93 g, KH_2_PO_4_ 2.54 g, 38% formaldehyde 125 mL, NaCl 5.41 g, distilled water qs 1000 mL) and smears were prepared according to Hancock [29] for morphological examination. At least 200 sperm were subsequently evaluated by phase contrast microscopy using oil immersion (Olympus BX50, UplanF1 100×/1.30, Olympus, Wallisellen, Switzerland). Morphologically abnormal sperm were assigned to defect categories according to Blom [30].

Sperm viability was assessed using the eosin-nigrosin staining method [31]. At least 200 sperm were evaluated on stained slides under oil immersion using a light microscope (Olympus BX50, UplanF1 100×/1.30, Olympus, Wallisellen).

The bull that was homozygous for the 1-bp deletion was slaughtered at 21 months at a regular slaughterhouse. Testis tissue was preserved in formalin, embedded in paraffin and subsequently stained with hematoxylin–eosin for histological evaluation.

### Transmission electron microscopy

Sperm were prepared for transmission electron microscopy (TEM) from fresh semen diluted in OptiXcell (IMV technologies, L’Aîgle, France) and stored at 5 °C overnight. Semen samples were washed twice by dilution in phosphate buffered saline (PBS) and subsequent centrifugation at 300*g* for 10 min. After removing the supernatant, the pellet was fixed with an equal volume of 6% glutaraldehyde in PBS, resuspended gently, and centrifuged at 6000*g*. After removing the supernatant, the sperm were fixed for a second time with 3% glutaraldehyde in PBS, and finally pelleted at 6000*g*. Pellets were washed three times in PBS, post-fixed in 1% osmium tetroxide, washed in double distilled water, stained in 1% uranyl acetate, dehydrated in a graded series of ethanol (25, 50, 75, 90, and 100%), and embedded in epoxy resin through increasing concentrations (25, 50, 75, and 100%) using PELCO BioWave Pro + tissue processor (Ted Pella, USA), and then cured at 60 °C for 3 days. Embedded blocks were sectioned using the Leica FC6 microtome and a DIATOME diamond knife at a 45° angle into 60-nm sections and mounted on Quantifoil copper grids with formvar carbon films. Sections were post-stained with 2% uranyl acetate followed by lead citrate. Grids were imaged using a FEI Morgagni 268 electron microscope operated at 100 kV at 8.9 to 56k magnification.

### Scanning electron microscopy

Sperm were prepared for scanning electron microscopy (SEM) within 30 min after semen collection. A thin layer of native semen was spread on carbon coated coverslips with the side of a pipet tip. The coverslips were immersed in 3% glutaraldehyde in PBS solution and kept on ice. After fixation overnight at 4 °C, fresh fixative was added and the sample was put in a PELCO BioWave Pro + microwave system (Ted Pella, USA). Following the microwave-assisted fixation and dehydration procedure, another fixation on ice was performed. After washing in PBS, samples were post-fixed in 1% OsO_4_ in double distilled water, washed again and dehydrated in a graded series of ethanol (50, 75, 90, 98, and three times 100%) on ice followed by critical point drying out of dry ethanol (CPD 931, Tousimis, USA). The resulting samples were mounted on SEM aluminium stubs, fixed on conductive carbon tape and then sputter-coated with 4 nm of platinum/palladium (CCU-10, Safematic, CH). Acquisition of images was performed using In-lens and Everhart–Thornley secondary electron (SE) signals at a working distance of 4 mm with a scanning electron microscope (Merlin FE-SEM, Zeiss, DE), operated at an accelerating voltage of 1.5 kV.

## Results

### Identification of a Brown Swiss bull with poor semen quality

We examined historic semen quality records from a semen collection centre in Switzerland as part of our ongoing efforts to investigate inherited variation in male reproduction in Brown Swiss bulls [1, 4, 32]. Among 1343 Brown Swiss bulls that produced 70,990 ejaculates, we identified seven bulls from which all ejaculates (between 5 and 28 per bull) were rejected due to immotile (asthenozoospermia), morphologically abnormal (teratozoospermia), low concentration (oligozoospermia), or absence of (azoospermia) sperm, or a combination of these anomalies. Because genetic material was not available for all these affected bulls, we focused on one of these bulls (born in 2003) from which preserved hair roots provided a source of DNA for genetic investigations.

Twelve ejaculates from this bull were collected between 15 and 18 months of age. The ejaculates had larger volumes (6.6 ± 1.5 vs. 4.7 ± 2.0 mL) and lower sperm concentrations (0.2 ± 0.2 vs. 1.3 ± 0.5 × 10^9^ sperm cells per mL) than observed in other Brown Swiss bulls of the same age. Almost all sperm (99%) were immotile and had head and tail abnormalities. The proportion of viable sperm was not recorded. Such findings are commonly referred to as oligoasthenoteratozoospermia.

### Candidate causal variants

The genome of the bull that produced ejaculates of insufficient quality was sequenced at 11.8-fold coverage using paired-end libraries (sequencing read archive accession: SAMEA6272098, thus this bull is referred to by this accession number). Its sequence variant genotypes were compared to those of 397 fertile artificial insemination bulls from various breeds that had an average sequencing coverage of 10.0 ± 5.7 fold (between 3.1- and 56.8-fold).

We hypothesized that the drastically reduced semen quality of SAMEA6272098 was due to a recessively inherited deleterious allele. From a catalogue of 41,659,308 autosomal sequence variants, we retained 1655 that were homozygous in SAMEA6272098 but not homozygous in the fertile control bulls (see Additional file 4: Table S4). Only three compatible variants were predicted to be deleterious to protein function: a nonsense variant in *MLNR* that encodes the motilin receptor, a frameshift variant in *QRICH2* that encodes the glutamine-rich protein 2, and a nonsense variant in *ENSBTAG00000023270* that encodes an uncharacterized protein (Table 1). Another nine (Ensembl) and ten (Refseq) compatible variants were predicted to have moderate impacts on the corresponding proteins.

**Table 1:** High impact variants compatible with recessive inheritance

Chr	Pos	Ref	Alt	Gene	Transcript abundance in testis (TPM)	Impact on protein	Ensembl/refseq/both
12	18,859,636	C	A	*MLNR*	0.07 ± 0.04	Cys587Ter	Both
19	55,436,705	TC	T	*QRICH2*	31.43 ± 7.35	Cys1644AlafsTer52	Both
24	10,181,719	G	A	*ENSBTAG00000023270*	0.04 ± 0.03	Trp248Ter	Ensembl

Chr: chromosome number; Pos: position of the variant on the genome; Ref: reference allele; Alt: alternate allele

In the testis transcriptomes from 76 mature bulls, *MLNR* and *ENSBTAG00000023270* were expressed with less than 0.1 transcripts per million (TPM), whereas *QRICH2* was transcribed in high abundance (31.43 TPM). Moreover, *QRICH2* shows an extremely testis-biased expression in mammals (e.g., https://gtexportal.org/home/, http://cattlegeneatlas.roslin.ed.ac.uk/) [33]. Pathogenic alleles of the human and mouse *QRICH2* gene cause sperm with multiple morphological abnormalities of the flagella [34–36]. Thus, we considered *QRICH2* as a compelling functional candidate gene for the sperm defect of SAMEA6272098 and pursued our analyses on this gene.

### A 1-bp deletion in the coding sequence of *QRICH2* is associated with aberrant semen quality

The candidate causal variant that underlies the sperm defect is a 1-bp deletion in exon 16 of *QRICH2* (at BTA19:55436705TC>T, ENSBTAT00000018337.1:c.4929del). This deletion of a cytosine residue is predicted to alter the reading frame from amino acid 1644 onwards, which results in a premature termination codon (ENSBTAP00000018337.1:p.Cys1644AlafsTer52). This leads to a truncated protein that lacks 131 amino acids (7%) from the C-terminal region unless the transcript is subjected to nonsense-mediated mRNA decay.

**Fig. 1:**
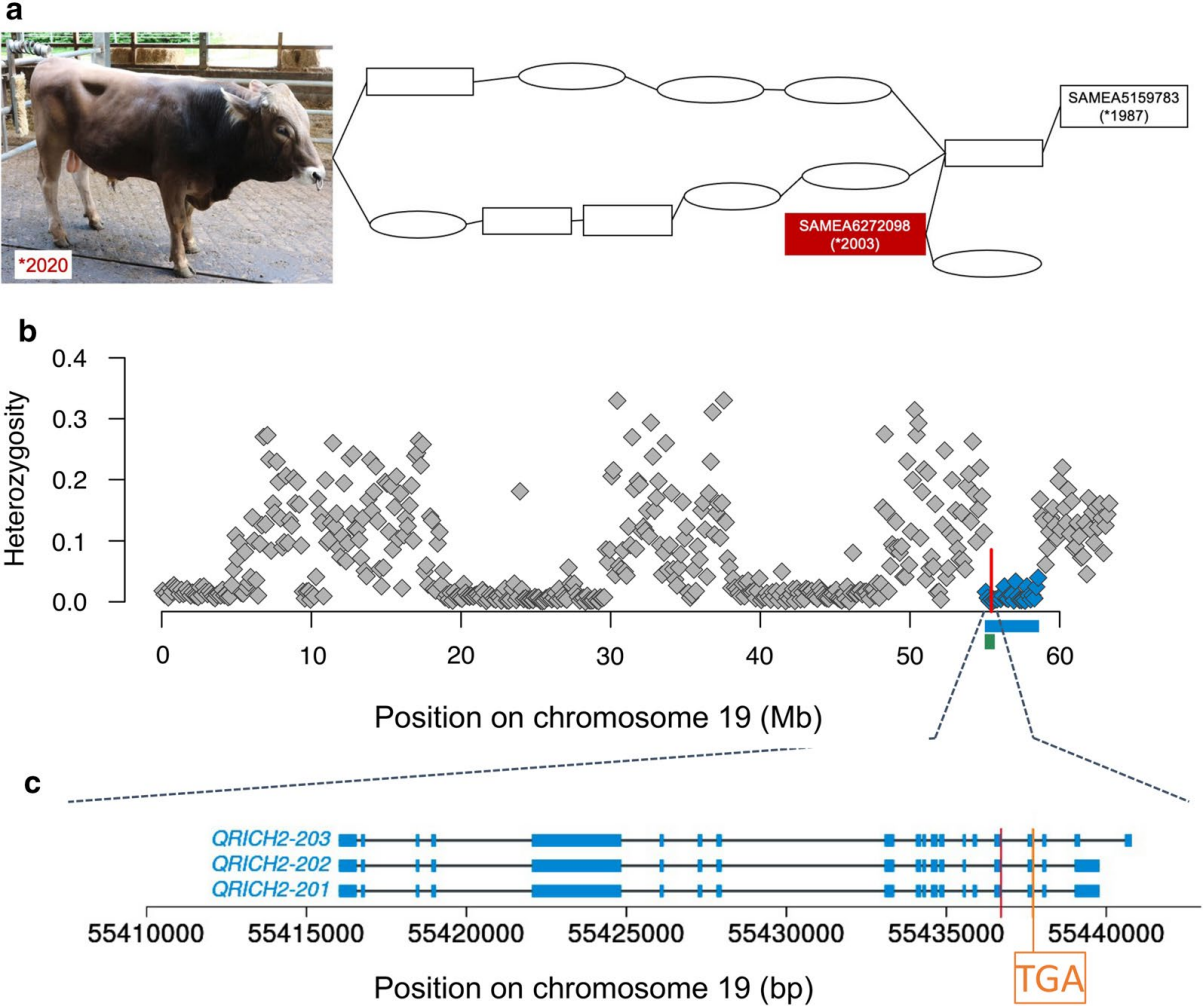
A 1-bp deletion is a candidate causal variant for a sperm morphology defect in Brown Swiss bulls. **a** Pedigree of two bulls with a defect of sperm heads and tails. The pedigree only contains suspected carriers of the 1-bp deletion. Ovals and rectangles represent cows and bulls, respectively. The bull born in 2020 and the SAMEA6272098 bull are related through a common ancestor. SAMEA5159783 is the oldest sequenced mutation carrier in the pedigree. **b** Each symbol represents the proportion of heterozygous genotypes observed within a 125-kb window for SAMEA6272098. Blue colour represents a 3.625-Mb segment of extended homozygosity encompassing BTA19:55436705TC>T (red vertical line). The green rectangle indicates the position of a BovineHD-based haplotype that encompasses the 1-bp deletion. **c** Structure of bovine *QRICH2* isoforms with transcript-ID ENSBTAT00000065208, ENSBTAT00000064147, and ENSBTAT00000018337 encoding proteins with 1968 (ENSBTAP00000055962), 1934 (ENSBTAP00000054965) and 1827 (ENSBTAP00000018337) amino acids. Blue rectangles represent exons. The red vertical line indicates the position of the 1-bp deletion (BTA19:55436705TC>T). Orange colour indicates the position of a premature termination codon (“TGA”) that is introduced due to the shift in translation caused by the 1-bp deletion

The pedigree-derived coefficient of inbreeding of the SAMEA6272098 bull is 7.61%. Due to this relatively high inbreeding, several extended segments of autozygosity are present in the genome of SAMEA6272098. The 1-bp deletion also resides within an approximately 3.625-Mb segment (between 55 and 58.625 Mb) of high homozygosity (Fig. 1). This pattern suggests that the 1-bp deletion resides in a haplotype that SAMEA6272098 inherited from an ancestor that is present both in its maternal and paternal ancestry.

Among 244 Brown Swiss and 124 Original Braunvieh cattle, which we had sequenced for previous projects and which are representative animals from both populations, we detected the 1-bp deletion in the heterozygous state in 25 Brown Swiss and 3 Original Braunvieh individuals, corresponding to an allele frequency of 5.1 and 1.2%, respectively. The heterozygous Original Braunvieh bulls were from Germany and we cannot rule out the possibility that the variant was recently introgressed through cross-breeding with Brown Swiss ancestors. The mutation did not segregate among 110 sequenced Original Braunvieh cattle from the Swiss herdbook population. We did not detect the mutation in breeds other than Brown Swiss and Original Braunvieh.

In a catalogue of variants that was established by the 1000 Bull Genomes Project consortium for 3934 cattle from various breeds, the 1-bp deletion was discovered in 16 animals from the Brown Swiss and one animal from the Nordic Red Dairy cattle breeds. The carrier animal from the Nordic Red Dairy cattle breed had an estimated proportion of 34% Brown Swiss ancestry. In the 1000 Bull Genomes dataset, only SAMEA6272098 carried the deletion in the homozygous state.

*QRICH2* mRNA was less abundant for six heterozygous carriers of the 1-bp deletion than for 70 bulls that were homozygous for the wild-type allele (23.29 ± 6.76 vs. 32.13 ± 7.01 TPM, P = 0.008, Wilcoxon test). Differential expression between heterozygous and wild-type bulls was evident (P < 0.05, Wilcoxon test) for 18 *QRICH2* exons (see Additional file 5: Fig. S1). We observed allelic imbalance at the 1-bp deletion in the total RNA sequencing alignments of all six heterozygous bulls; an average of 292 ± 143 sequencing reads overlapped with the BTA19:55436705TC>T position, but less than a third (81 ± 32) supported the deletion. Across all the heterozygous bulls, 1266 and 487 mapped reads supported the reference and alternate allele, respectively (P = 9.45 × 10^–80^, binomial test (see Additional file 5: Fig. S1)), indicating that the RNA of heterozygous bulls is depleted for the *QRICH2* transcript carrying the deletion.

### Identification of homozygous haplotype carriers in the Brown Swiss population

To validate the hypothesis that the sperm defect was caused by the 1-bp deletion, we examined ejaculates from homozygous bulls. Because SAMEA6272098 had been slaughtered in 2005 and no semen samples had been preserved, it was not possible to re-examine its sperm defect. In order to identify homozygous carriers for this mutation, we used a dataset that contained 25,243 Brown Swiss and 5228 Original Braunvieh cattle with phased Illumina BovineHD Bead chip genotypes at 17,579 SNPs located on BTA19. These animals were genotyped for routine genomic breeding value prediction in Switzerland. Of the 30,471 genotyped Brown Swiss and Original Braunvieh cattle, 285 (28 females, 257 males) also had whole-genome sequence-called genotypes at the BTA19:55436705TC>T variant. Twenty-one sequenced animals (1 female, 20 males) were heterozygous carriers of the 1-bp deletion. Under the assumption that these 21 animals inherited the 1-bp deletion from a common ancestor, we screened their phased array-derived genotypes for a shared haplotype encompassing BTA19:55436705TC>T. This approach identified a 675-kb haplotype that contained 181 Illumina BovineHD BeadChip SNPs and spanned from 54,992,461 to 55,667,539 bp on BTA19 and that was present in the heterozygous state in the 21 heterozygous animals (Fig. 1 and see Additional file 2: Table S2).

Of the 264 sequenced animals that did not carry the 1-bp deletion, only one animal (SAMEA7573647) carried the haplotype in the heterozygous state. This animal was a fertile paternal half-sib of SAMEA6272098. Upon manual inspection of the read alignments, we noticed that only one properly mapped sequencing read overlapped with BTA19:55436705TC>T in the sequence of the SAMEA7573647 bull (see Additional file 6: Fig S2). This read supported the 1-bp deletion, although SAMEA7573647 was genotyped as homozygous for the reference cytosine residue. Low-sequencing coverage likely resulted in undercalling of heterozygous genotypes. Thus, the microarray-derived haplotype was in full concordance with the genotypes at the BTA19:55436705 TC>T variant in the 285 sequenced animals.

The 675-kb haplotype had a frequency of 5.4% among 25,243 genotyped Brown Swiss cattle. This haplotype frequency does not appear to have changed notably over the past 30 years. We detected 2712 heterozygous and 54 homozygous haplotype carriers, whereas 79 homozygous animals were expected assuming random mating, thus the distribution of the observed haplotypes deviated slightly from the Hardy–Weinberg proportions (P = 0.03). The maternal grandsire from the heterozygous bull from the Nordic Red Dairy Cattle breed carried the haplotype in the heterozygous state. Among the homozygous haplotype carriers, 35 were females and 19 were males. Since none of the homozygous bulls had been selected as an artificial insemination sire, their semen quality was not examined. Thirty-three of the 35 homozygous females gave birth to at least one offspring, indicating that their fertility was not compromised. Inspection of uncorrected milk records in heterozygous cows indicated that their milk production is normal. None of the 5228 Original Braunvieh cattle carried the 675-kb haplotype.

We used semen quality data from 1142 Brown Swiss bulls that were previously analyzed by Hiltpold et al. [1, 4] to investigate if the 675-kb haplotype is associated with semen quality in the heterozygous state. We examined an average number of 39 (between 5 and 186) and 41 (between 1 and 549) ejaculates from 143 heterozygous haplotype carriers and 999 bulls that did not carry the haplotype, respectively. The absolute values for ejaculate volume (in mL), sperm concentration (million sperm per mL), and percentage of motile sperm were within reference ranges for both heterozygous carriers (3.99 ± 1.16, 1270 ± 395, and 83 ± 11) and non-carriers of the haplotype (4.22 ± 1.21, 1234 ± 328, and 83 ± 9). The differences in semen quality between carriers and non-carriers of the haplotype were only statistically significant for ejaculate volume (P = 0.017, Wilcoxon test). However, mixed model-based association testing between semen quality and dense markers did not reveal any QTL for semen quality in this genomic region [32]. Thus, we assume that the observed difference in ejaculate is due to factors other than the 1-bp deletion. The proportion of ejaculates that were rejected or contained an excess of sperm with head and tail anomalies did not differ notably between heterozygous carriers and non-carriers, supporting a recessive mode of inheritance.

Since all the male homozygous carriers of the haplotype had been slaughtered before our study was conducted, no ejaculate collection and semen analyses could be performed. In order to identify homozygous bulls for phenotypic investigations, the coordinates of the 675-kb haplotype were sent to the Brown Swiss genetic evaluation centers in Switzerland, Austria and Germany. In 2020, a haplotype screen of the genomic selection reference populations identified a 6-month-old bull that carried the haplotype in the homozygous state. The sire of SAMEA6272098 was in the maternal (6th ancestral generation) and paternal (5th ancestral generation) ancestry of the 6-month-old homozygous haplotype carrier indicating inbreeding due to an obligate carrier of the 1-bp deletion (Fig. 1a). Sanger sequencing confirmed homozygosity for the 1-bp deletion (BTA19:55436705TC>T). The bull was apparently regularly developed and healthy.

### Ejaculates of a homozygous bull contain immotile sperm with multiple morphological abnormalities

We kept the bull that was homozygous for the 1-bp deletion at a research station together with other bulls of similar age. At 14 months, its scrotal circumference was relatively small (31 cm) indicating delayed puberty. The first ejaculate collected from the bull had a volume of 5 mL, but it contained only 0.03 × 10^9^ sperm per mL (Table 2). None of the sperm were motile and 97.3% had tail or head abnormalities. The scrotal circumference increased as the bull grew older (34.5 cm at 20 months) but remained relatively small compared to that of the other bulls. We collected six additional ejaculates from the bull at 16, 17, and 20 months. The ejaculates had an average volume of 4.0 ± 0.9 mL and contained 0.2 ± 0.1 × 10^9^ sperm per mL, confirming a normal volume but an aberrantly low sperm concentration and sperm count. More than 99% of the sperm had major defects and less than 1% were motile. Histological examination of the testis after slaughter indicated that Leydig cells, rete testis, and tubuli seminiferi were formed normally and spermatogenesis was detectable.

**Table 2:** Semen analysis in a bull homozygous for the 1-bp deletion in the coding sequence of *QRICH2*

Age of the bull (months)	Scrotal circumference (cm)	Ejaculate volume (mL)	Sperm concentration (10^9^ sperm per mL)	Sperm with major defects (%)	Motile sperm (%)	Viable sperm (%)
14	31	5.0	0.031	97.3	0	na
16	32.5	4.0	0.187	99.6	< 1	na
		5.0	0.115	97.9	< 1	na
17	33.5	2.8	0.160	100	0	12
		3.0	0.088	98.7	< 1	10.8
20	34.5	4.5	0.214	99.2	0	4.2
		4.5	0.360	99.3	< 1	10.4

*na* not analysed

**Fig. 2:**
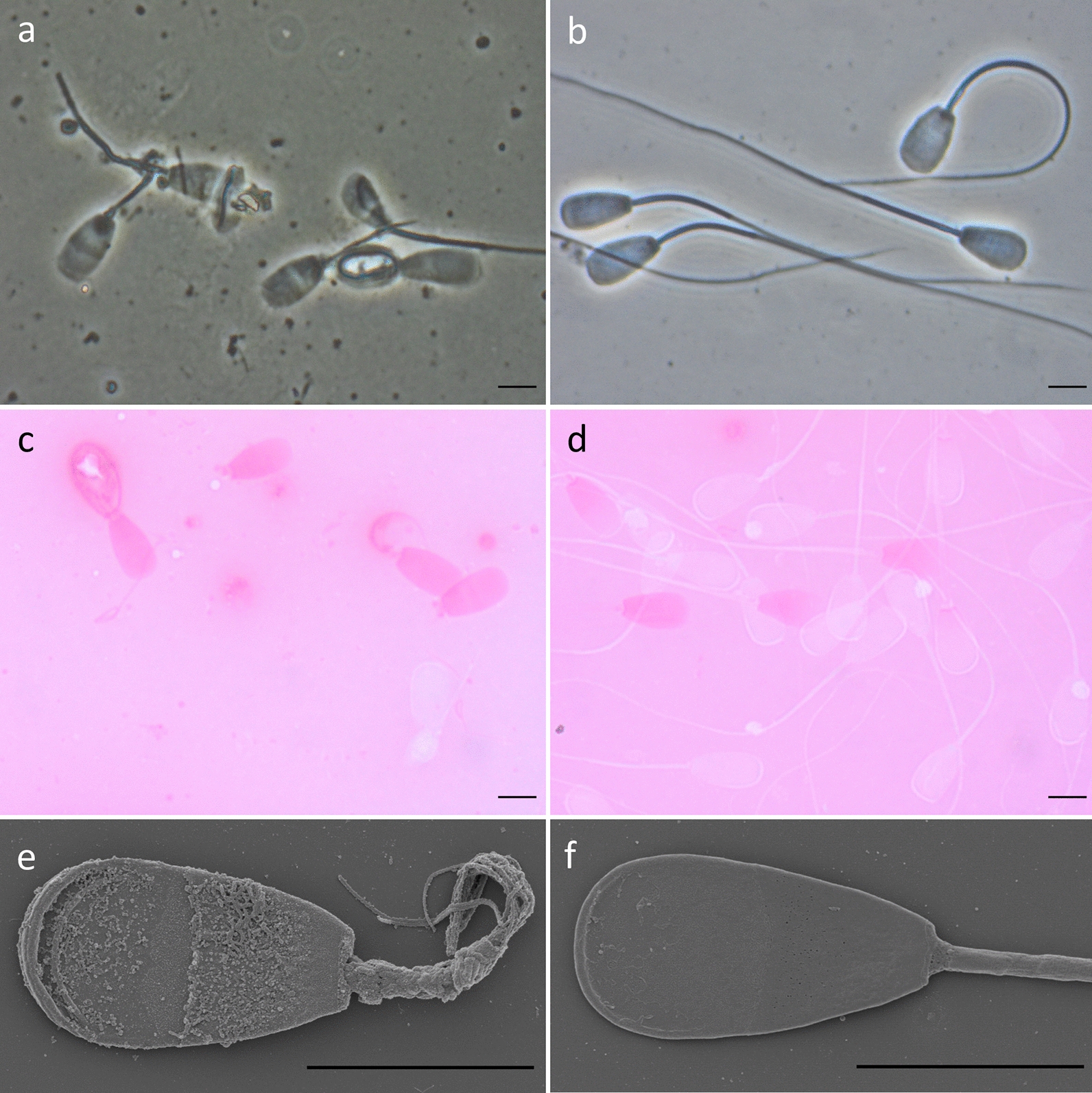
Sperm of a bull homozygous for the 1-bp deletion and of a control bull. Representative phase-contrast (**a**, **b**), eosin-nigrosin stained (**c**, **d**), and scanning electron microscopy images (**e**, **f**) of sperm of a bull homozygous for the 1-bp deletion (**a**, **c**, **e**) and of a control bull (**b**, **d**, **f**) with normal sperm quality. Sperm of the affected bull show multiple morphological abnormalities of head and flagella (**a**). Phase-contrast microscopy also revealed numerous cell debris particles. Viable sperm remain unstained and appear white in eosin-nigrosin stained images whereas dead sperm are stained and appear purple (**c**, **d**). Flagella of the affected bull are irregularly shaped and mostly have an uneven surface (**e**). Scale bar: 5 µm

Microscopic analysis of the semen revealed that almost all the sperm had multiple morphological abnormalities that primarily affected the flagella (Fig. 2 and see Additional file 7: Fig. S3). Morphological abnormalities included shortened, coiled, bent, looped, thickened, doubled and absent tails. Loose tails were not detected. Approximately 20% of the examined sperm were underdeveloped, had irregularly shaped heads, or nuclear vacuoles. Eosin-nigrosin staining indicated that between 4 and 12% of the sperm were viable in spite of morphological abnormalities of the head and flagella (Fig. 2c).

**Fig. 3:**
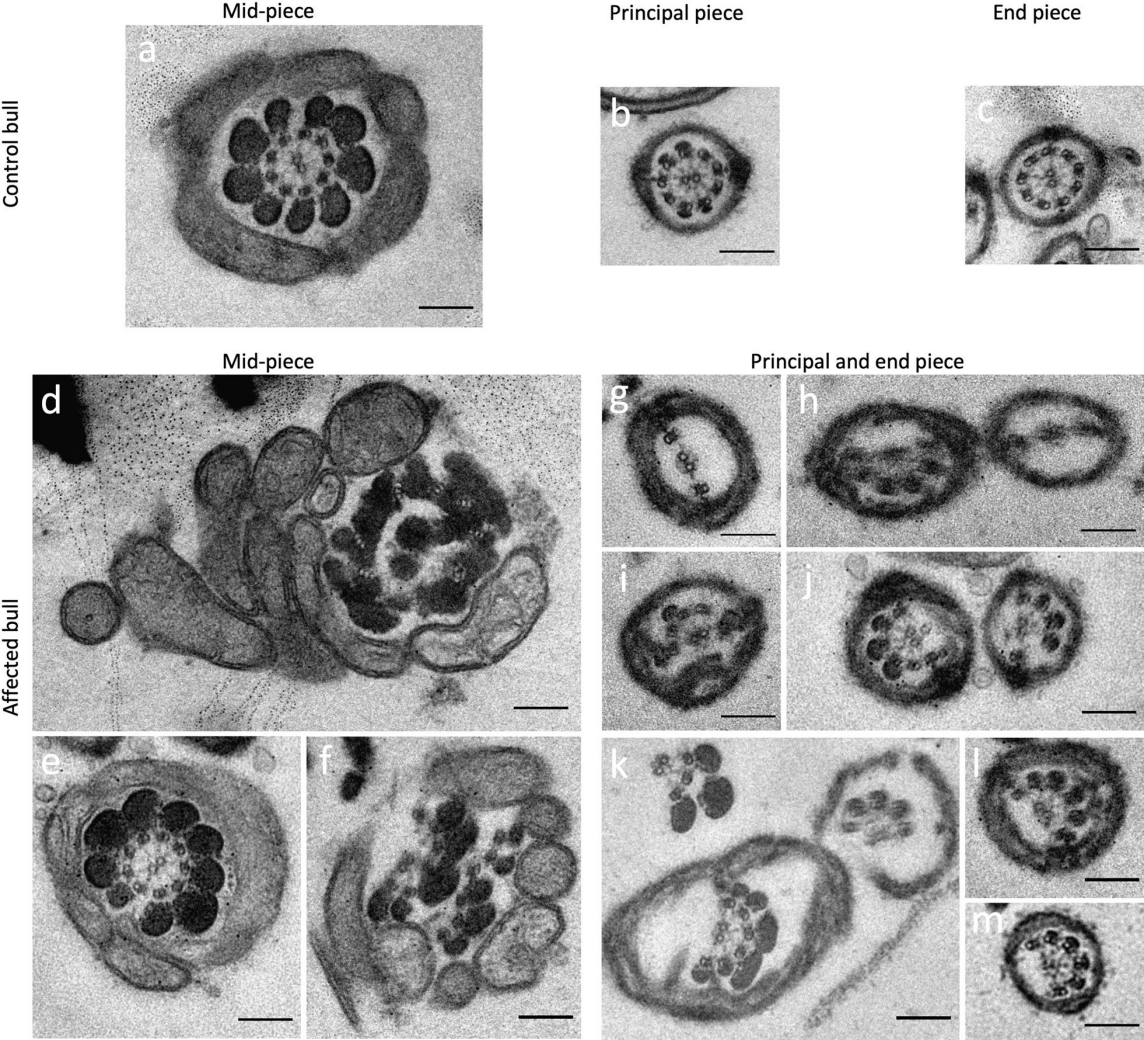
Transmission electron microscopy (TEM) cross-sections of bovine sperm flagella. Representative TEM cross-sections of sperm flagella at the mid-piece, principal piece and end piece in a control bull (**a**–**c**) and a bull homozygous for the 1-bp deletion in *QRICH2* (**d**–**m**). Irregularities in the flagellar cross-sections from the homozygous bull prevent unambiguous assignment of the principal and end piece (**g**–**m**). The axonemes of most of the flagella lack some of the outer microtubules resulting in deviations from the typical arrangement of nine outer microtubule doublets surrounding the central pair in the homozygous bull. Cross-sections also show irregular assembly of axonemes and structures such as outer dense fibres that are supposed to enclose the axonemes (**d**, **f**). Scale bar: 200 nm

Transmission electron microscopy cross-sections of sperm from the affected bull confirmed multiple ultrastructural defects of the flagellum and head (Fig. 3 and see Additional file 8: Fig. S4). The axonemes of most flagella deviated from the typical assembly of nine outer microtubule doublets surrounding the central pair. A variable number of outer microtubule doublets was missing in the principal and end piece region of most of the examined flagella. We also detected sperm with multiple flagellar structures within the same cell membrane, indicating that the flagellum was folded around the head instead of being elongated which is typical for underdeveloped sperm (Additional file 8: Fig. S4). Occasionally, we found axonemes in cross-sections of the principal piece that appeared regularly organized.

## Discussion

In this paper, we describe the detection of a recessive 1-bp deletion inducing a frameshift in the *QRICH2* gene in two Brown Swiss bulls producing ejaculates with low sperm concentration and immotile sperm cells with multiple morphological abnormalities. Semen samples from these bulls were not used for artificial inseminations because the resulting disorder very likely prevents fertilization in vivo due to the inability of the sperm to move. Intracytoplasmic sperm injection (ICSI)—although not a common practice in cattle breeding—could possibly enable fertilization because some sperm were viable and had normally shaped heads. In humans, fertilization and normal offspring development were obtained with ICSI using sperm with multiple morphological abnormalities of the flagella that were caused by deleterious variants in *QRICH2* [36].

We applied an indirect haplotype test and found that the 1-bp deletion segregates at a frequency of 5% in the Brown Swiss population. Assuming random mating, the deletion is expected to occur in the homozygous state in 1 out of 400 bulls. However, the proportion of homozygous bulls was lower in our study. None of the 1142 Brown Swiss bulls for which we had microarray-derived genotypes and routinely assessed semen quality phenotypes, carried the haplotype in the homozygous state. The 1-bp deletion resides in a haplotype that is inherited from a common ancestor. The mating of closely related individuals is generally avoided to prevent inbreeding and therefore haplotypes may occur less frequently in the homozygous state than expected under random mating. In principle, the frequency of the 1-bp deletion may be lower than estimated from an indirect haplotype test. If a mutation occurred relatively recently, the haplotype encompassing the mutation may be indistinguishable from its ancestral form that does not carry the mutation. In such a situation, a haplotype-based test overestimates the frequency of the mutation [37]. However, we found no evidence that an ancestral version of the haplotype segregates in the Brown Swiss population, as the 675-kb haplotype was in full concordance with the 1-bp deletion. Moreover, the frequency of this haplotype agreed well with the frequency of the 1-bp deletion estimated from whole-genome sequence variants.

The birth years of the two homozygous bulls span almost two decades. Only one of these bulls was selected as a potential artificial insemination sire. Since immotile sperm with multiple morphological abnormalities were detected during routine semen analyses, all ejaculates from this bull were discarded and not used for artificial inseminations. Heterozygous bulls produce normal sperm. If the semen of prospective artificial insemination and natural mating bulls is examined, this sperm defect is easily recognized and thus will not manifest itself in repeated breedings and low non-return rates as is the case for infertility disorders that are not detected from standard semen analyses [8]. Economic losses due to this defect are negligible in the Brown Swiss cattle population because the frequency of the 1-bp deletion is low and aberrant semen quality of homozygous bulls can be easily detected.

The widespread use of individual carriers of this mutation in artificial insemination can result in the occurrence of frequent recessive alleles within a short time [38]. It is advised to monitor the BTA19:55436705TC>T variant in the Brown Swiss population, e.g., by using customized microarrays, and to implement genome-based mating programs to avoid the frequent emergence of bulls that produce ejaculates containing immotile sperm. We did not detect the BTA19:55436705TC>T variant in Swiss Original Braunvieh animals, which suggests that the mutation occurred relatively recently possibly after the separation of the Braunvieh breed into the dairy (Brown Swiss) and dual-purpose (Original Braunvieh) lineages. We found the 1-bp deletion in one animal from the Nordic Red Dairy Cattle breed, which likely resulted from crossbreeding with a heterozygous Brown Swiss sire [39]. Thus, our results increase the number of known alleles with undesired effects that have been introgressed from foreign breeds into the Nordic Red Dairy Cattle breed [40–42].

Human and mouse orthologs of the *QRICH2* gene harbour loss-of-function variants that result in multiple morphological abnormalities and ultrastructural defects of the sperm flagella, thereby leading to immotile sperm and infertility in vivo [34, 35, 43]. We have replicated these results by analyzing the ejaculates from two bulls that are homozygous for a frameshift-inducing 1-bp deletion in the bovine *QRICH2* gene, which suggests an evolutionarily conserved protein function. The 1-bp deletion is predicted to induce a premature termination codon that shortens the protein by 131 amino acids. The truncated protein may be retained with reduced functionality or the transcript may be degraded via nonsense-mediated mRNA decay. The position of the premature termination codon complies with both canonical and non-canonical rules for nonsense-mediated mRNA decay [44–46]. Allelic imbalance at the 1-bp deletion and the lower levels of *QRICH2* mRNA in the testis transcriptomes of heterozygous than in those of wild-type bulls confirm that the premature termination codon triggers nonsense-mediated mRNA decay. These findings show that the 1-bp deletion constitutes a loss-of-function allele of the bovine *QRICH2* gene, thereby supporting the pathogenicity of a predicted deleterious variant that resides in a similar domain of the human *QRICH2* gene [35].

To date, the QRICH2 protein has primarily been implicated in flagellar formation of the sperm. Apart from flagellar abnormalities, we observed sperm head anomalies, drastically reduced sperm concentration and low sperm count in all the ejaculates examined from bulls homozygous for the 1-bp deletion. Sperm concentration and total sperm count were also at the lower bound of the normal range in humans with deleterious *QRICH2* alleles [34, 35, 43]. These findings suggest that loss of QRICH2 functionality not only compromises the assembly of sperm flagella, but generally impairs spermiogenesis. Variants other than the 1-bp deletion may contribute to the aberrant semen quality of the two examined Brown Swiss bulls. However, pinpointing putative modifier loci is difficult for rare alleles [47] and was not attempted for the 1-bp deletion in *QRICH2*.

To the best of our knowledge, this is the first time that an effect arising from a loss-of-function allele in *QRICH2* is described in a species other than mice and humans. Apart from producing anomalous ejaculates, homozygous bulls are indistinguishable from wild-type and heterozygous individuals. Homozygous cows were not examined clinically in our study, but their normal fertility and milk production likely indicate an overall normal health [48]. Moreover, no QTL for economically relevant traits have been detected next to BTA19:55436705TC>T in Brown Swiss cattle [49]. These findings suggest that the 1-bp deletion does not compromise traits other than semen quality. This agrees with loss-of-function alleles in other genes with extreme testis-biased expression [8–10, 50] and corroborates findings in humans and mice with loss-of-function alleles in *QRICH2* [34], which suggest that QRICH2 is not essential for somatic development in mammals. The deviation from Hardy–Weinberg proportions (P = 0.03) of the haplotype encompassing the 1-bp deletion is likely due to either selective breeding or the inability of homozygous males to contribute to the next generation rather than to fatal consequences arising from homozygosity.

Our analyses in two homozygous bulls provide evidence for the causality of the 1-bp deletion from a statistical, functional, and comparative genomics point of view [51, 52]. We discovered the deletion using a phenotype-driven approach in a historic animal sample and verified its phenotypic consequences in a second bull that was born almost two decades later and was identified through a genotype-driven screen. Transcriptome analyses show that the 1-bp deletion triggers nonsense-mediated mRNA decay, which confirms that it constitutes a loss-of-function allele. Orthologs of the bovine *QRICH2* gene harbour loss-of-function alleles; some of them reside in the same domain as the 1-bp deletion identified in our study and cause sperm defects mirroring those of the homozygous Brown Swiss bulls, which suggests that QRICH2 is essential for male fertility in mammals. Although a formal proof for the causality remains to be produced, these pieces of evidence are sufficient to warrant the monitoring of the 1-bp deletion in cattle breeding programs.

## Conclusions

A 1-bp deletion in the coding sequence of the bovine *QRICH2* gene is likely a causal mutation for low sperm concentration and immotile sperm with multiple morphological abnormalities in the homozygous state. The 1-bp deletion has a frequency of 5% in the Brown Swiss cattle population. Homozygous bulls are unsuitable for breeding as they are very likely infertile in vivo. Apart from having a poor semen quality, these homozygous bulls are indistinguishable from heterozygous and wild-type animals. Although the immediate economic consequences due to the undesired allele are negligible, the monitoring of the defective allele in the Brown Swiss cattle population using customized genotyping is recommended to avoid increasing its frequency.

## Supplementary Information

**Additional file 1: Table S1.** Sequence read archive accession numbers for the sequenced animals. The spreadsheet file contains accession numbers from the European Nucleotide Archive that point to sequencing data for cattle from various breeds. The data in the file are organized in three spreadsheets. The accession for the bull that produced ejaculates with poor quality is listed in «case_SRA_ID». Accessions for 397 fertile control animals from various breeds are listed in «controls_SRA_IDs». Accessions, sequence-based genotypes and haplotypes for 285 cattle used to assign the 1-bp deletion onto a haplotype are listed in «array-based genotypes».

**Additional file 2: Table S2.** Coordinates of a diagnostic haplotype. Illumina BovineHD coordinates (SNP-name, chromosome, physical position [ARS-UCD1.2]) and haplotype allele of 181 markers that define the diagnostic 675-kb haplotype.

**Additional file 3: Table S3.** Accessions for the eQTL cohort. Accessions for sequencing reads obtained from RNA and DNA prepared from testis tissue of 76 mature bulls.

**Additional file 4: Table S4.** Candidate causal variants. Functional consequences predicted for 1655 variants that are compatible with recessive inheritance based on the Ensembl (version 104) and Refseq (version 106) annotation of the bovine genome.

**Additional file 5: Figure S1.** *QRICH2* mRNA analysis. (a) Exon-specific expression (quantified in transcripts per million [TPM]) for *QRICH2* in the testis tissue of six heterozygous (green) and 70 homozygous wild-type (grey) bulls. Red asterisks indicate exons that were differentially expressed (P < 0.05, Wilcoxon rank sum test) between heterozygous carriers and wild type bulls. (b) Integrative Genomics Viewer coverage tracks from RNA sequence read alignments overlapping the frameshift-inducing 1-bp deletion (red arrow) in six heterozygous bulls. The identifiers of the coverage tracks refer to accessions from the sequence read archive.

**Additional file 6: Figure S2.** DNA sequence alignment of SAMEA6272098. Output from «samtools tview» centered on BTA19:55436705TC>T representing DNA sequence read alignments from a bull (SAMEA6272098) that carries the 675-kb haplotype in the heterozygous state, but was genotyped as homozygous for the reference allele at the position of the BTA19:55436705TC>T variant. The asterisk within the only read that overlaps BTA19:55436706 indicates that the bull carries the 1-bp deletion.

**Additional file 7: Figure S3.** Phase-contrast images of sperm from a bull homozygous for the 1-bp deletion. All images display sperm with major sperm head and/or flagellar defects. Very short, shortened or absent flagella were the most prevalent flagellar abnormalities: very short flagella (a, b, d, e, f, g, i, k, s, u), shortened flagella (c, e, h, i, l, q, r,), and absent flagella (c, i, j, o, t). Short doubled (e, k, s) or short thickened (e, k, i, n, q) were apparent too. Some flagella were strongly folded (m, y right sperm) or coiled (y left sperm). Many sperm showed defective heads as well as defective flagella: pyriform (c, i, m, s, t), round (g, r), abnormal contour (p), and diadem defect/vacuoles (b, o, q, u, y). Underdeveloped sperm with the flagella strongly folded around the sperm head are considered the most severe sperm defects in bull (d right sperm, t right sperm, v, w, x). Scale bar: 5 µm.

**Additional file 8: Figure S4.** TEM and SEM images of sperm from a bull homozygous for the 1-bp deletion. TEM cross-section of an underdeveloped sperm with multiple flagellar structures next to the nucleus (black) enclosed by a cell membrane (a). Underdeveloped sperm with the flagellum curled on the head visualized using SEM (b). Longitudinal TEM cross-section (c) and SEM (d) of sperms with a thickened vesicularized structure at the sperm neck and multiple disorganized flagellar structures at the mid-piece. Longitudinal TEM cross-section of the neck region of a normal sperm from a control bull (e). Scale bar: 1 µm.

## Data Availability

Whole-genome sequencing data of 397 fertile bulls and the infertile bull are available at the European Nucleotide Archive (ENA) of the EMBL under sample accessions listed in Additional file 1: Table S1. Whole-genome sequencing data of the 285 cattle used to identify the trait-associated haplotype are available at the European Nucleotide Archive (ENA) of the EMBL under sample accessions listed in Additional file 1: Table S1. DNA and RNA sequencing data of the 76 bulls in the eQTL cohort are available at the European Nucleotide Archive (ENA) of the EMBL under sample accessions listed in Additional file 3: Table S3.
