# Supplementary figures and images for: A 1-bp deletion in bovine QRICH2 causes low sperm count and immotile sperm with multiple morphological abnormalities
Source: Genet Sel Evol. 2022 Mar 7;54:18. doi: 10.1186/s12711-022-00710-0 (PMC8900305; doi:10.1186/s12711-022-00710-0)

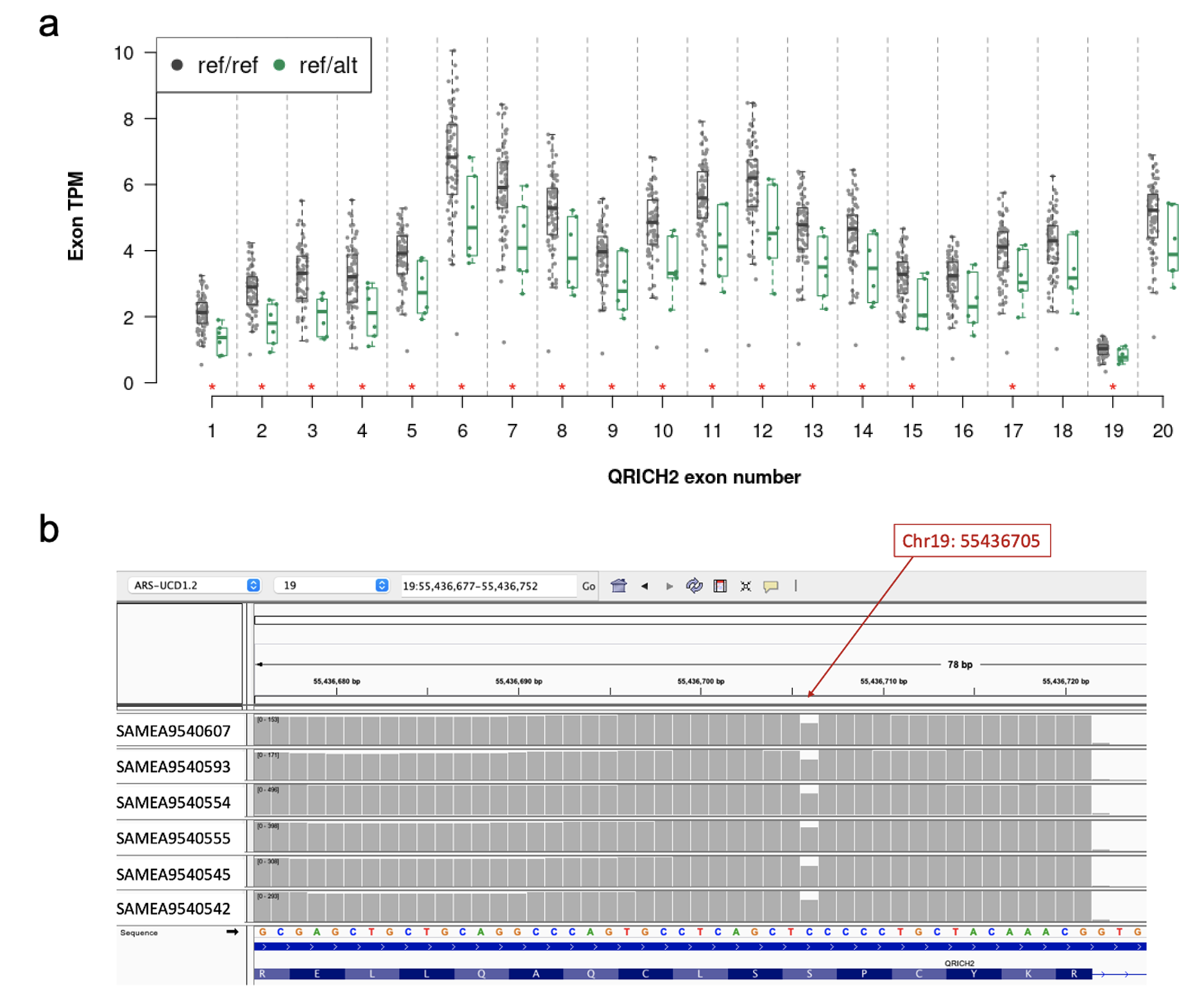

Supplement: Supplementary file 5 — Additional file 5: Figure S1. QRICH2 mRNA analysis. (a) Exon-specific expression (quantified in transcripts per million [TPM]) for QRICH2 in the testis tissue of six heterozygous (green) and 70 homozygous wild-type (grey) bulls. Red asterisks indicate exons that were differentially expressed (P < 0.05, Wilcoxon rank sum test) between heterozygous carriers and wild type bulls. (b) Integrative Genomics Viewer coverage tracks from RNA sequence read alignments overlapping the frameshift-inducing 1-bp deletion (red arrow) in six heterozygous bulls. The identifiers of the coverage tracks refer to accessions from the sequence read archive. [file 12711_2022_710_MOESM5_ESM.png]

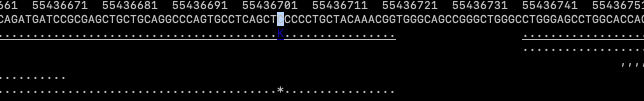

Supplement: Supplementary file 6 — Additional file 6: Figure S2. DNA sequence alignment of SAMEA6272098. Output from «samtools tview» centered on BTA19:55436705TC>T representing DNA sequence read alignments from a bull (SAMEA6272098) that carries the 675-kb haplotype in the heterozygous state, but was genotyped as homozygous for the reference allele at the position of the BTA19:55436705TC>T variant. The asterisk within the only read that overlaps BTA19:55436706 indicates that the bull carries the 1-bp deletion. [file 12711_2022_710_MOESM6_ESM.png]

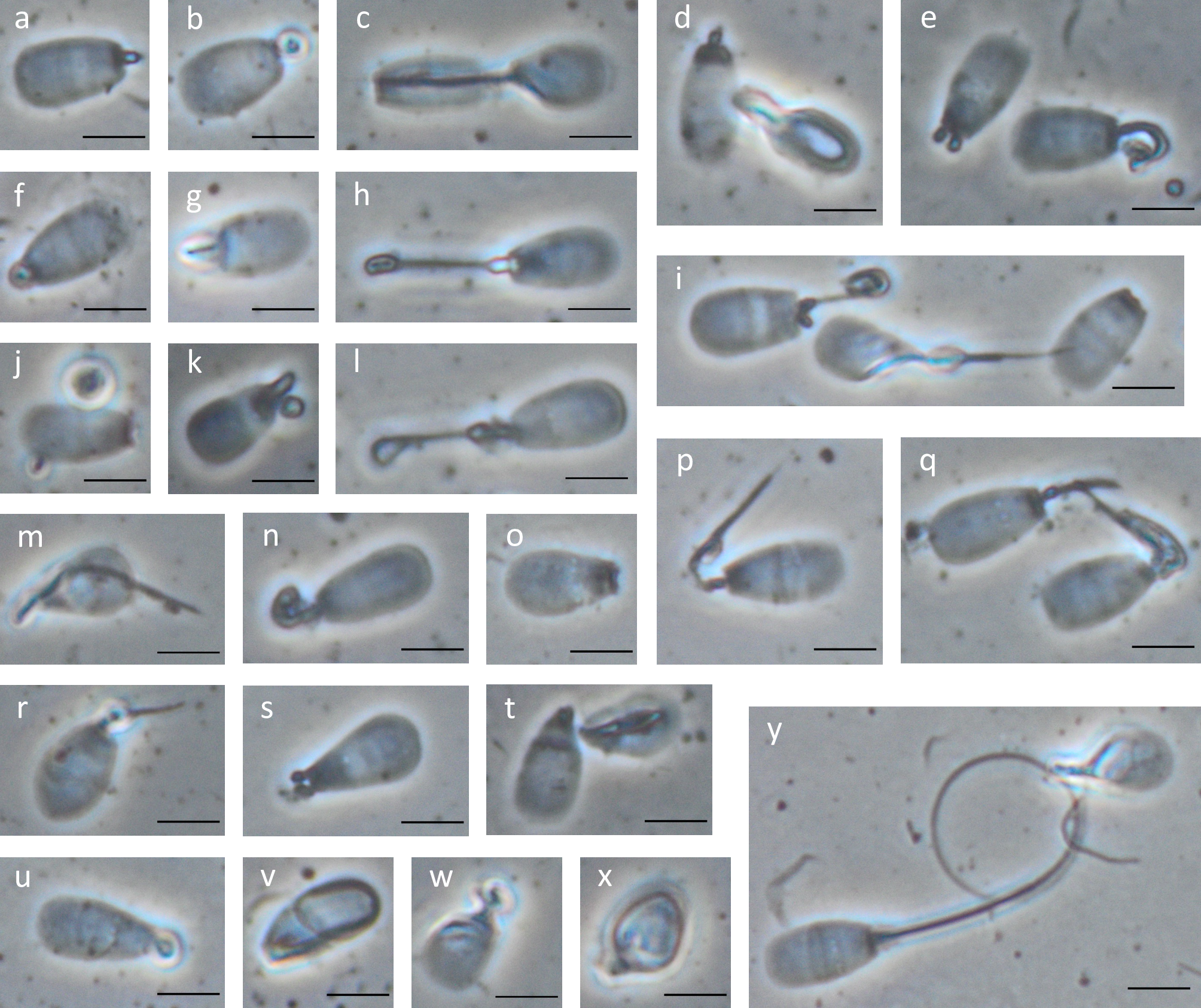

Supplement: Supplementary file 7 — Additional file 7: Figure S3. Phase-contrast images of sperm from a bull homozygous for the 1-bp deletion. All images display sperm with major sperm head and/or flagellar defects. Very short, shortened or absent flagella were the most prevalent flagellar abnormalities: very short flagella (a, b, d, e, f, g, i, k, s, u), shortened flagella (c, e, h, i, l, q, r,), and absent flagella (c, i, j, o, t). Short doubled (e, k, s) or short thickened (e, k, i, n, q) were apparent too. Some flagella were strongly folded (m, y right sperm) or coiled (y left sperm). Many sperm showed defective heads as well as defective flagella: pyriform (c, i, m, s, t), round (g, r), abnormal contour (p), and diadem defect/vacuoles (b, o, q, u, y). Underdeveloped sperm with the flagella strongly folded around the sperm head are considered the most severe sperm defects in bull (d right sperm, t right sperm, v, w, x). Scale bar: 5 µm. [file 12711_2022_710_MOESM7_ESM.tif]

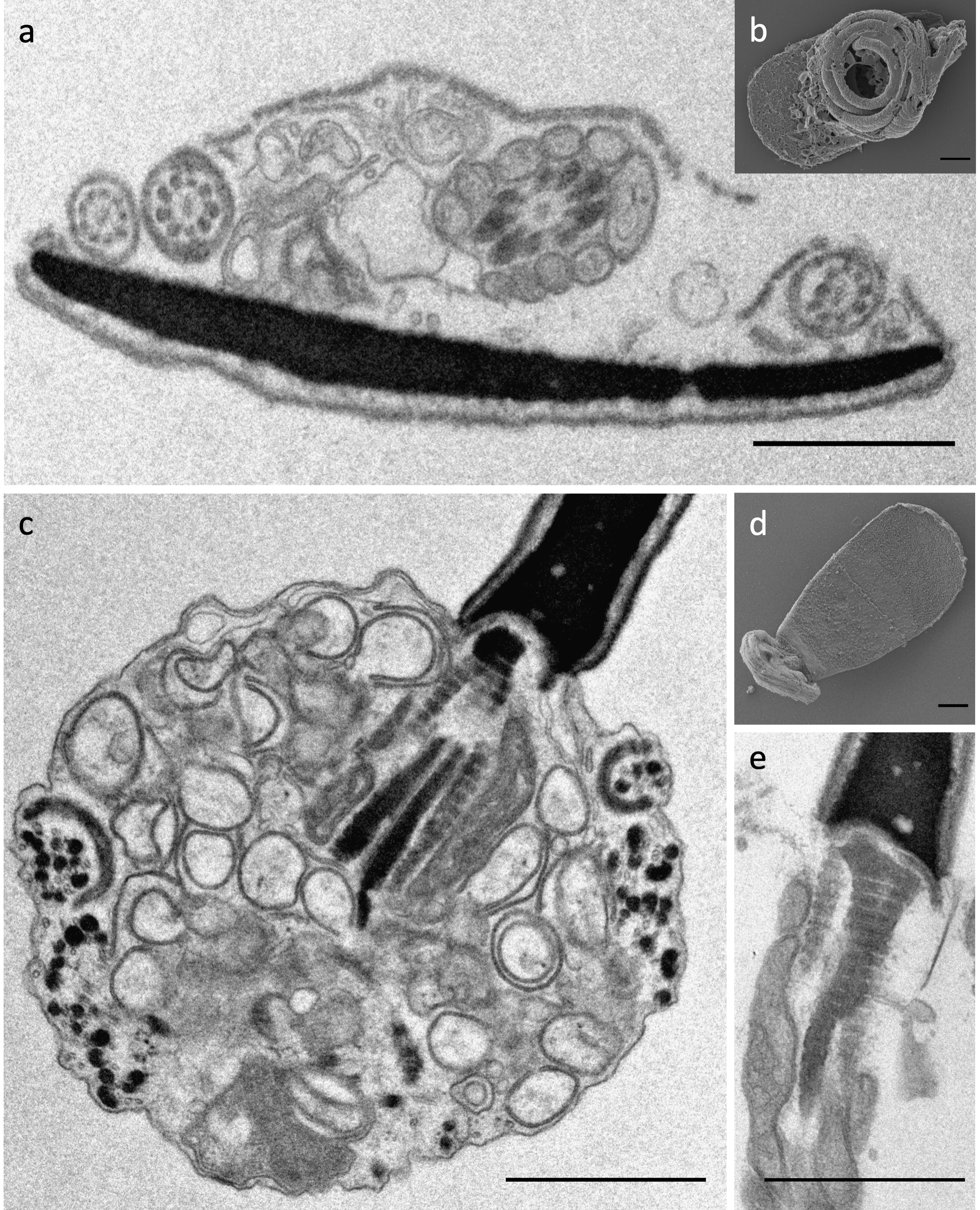

Supplement: Supplementary file 8 — Additional file 8: Figure S4. TEM and SEM images of sperm from a bull homozygous for the 1-bp deletion. TEM cross-section of an underdeveloped sperm with multiple flagellar structures next to the nucleus (black) enclosed by a cell membrane (a). Underdeveloped sperm with the flagellum curled on the head visualized using SEM (b). Longitudinal TEM cross-section (c) and SEM (d) of sperms with a thickened vesicularized structure at the sperm neck and multiple disorganized flagellar structures at the mid-piece. Longitudinal TEM cross-section of the neck region of a normal sperm from a control bull (e). Scale bar: 1 µm. [file 12711_2022_710_MOESM8_ESM.tif]
